# Social Determinants of Health and Antibiotic Consumption

**DOI:** 10.3390/antibiotics14050513

**Published:** 2025-05-15

**Authors:** Yuliya Semenova, Kamila Akhmetova, Daniil Semenov, Larissa Makalkina, Vladimir Surov, Lyudmila Pivina, Assiya Turgambayeva, Tatiana Belikhina, Saule Maukayeva, Maya Goremykina, Praveen Kumar

**Affiliations:** 1 Department of Surgery, School of Medicine, Nazarbayev University, Astana 010000, Kazakhstan; yuliya.semenova@nu.edu.kz; 2 Department of Public Health and Management, Astana Medical University, Astana 010000, Kazakhstan; turgambaeva.a@amu.kz; 3 Department of Computer Engineering, Astana IT University, Astana 010000, Kazakhstan; daniil.semenov@nu.edu.kz (D.S.); praveen.kumar@astanait.edu.kz (P.K.); 4 Department of Clinical Pharmacology, Astana Medical University, Astana 010000, Kazakhstan; makalkina.l@amu.kz; 5 Department of Biomedicine, School of Medicine, Nazarbayev University, Astana 010000, Kazakhstan; vladimir.surov@nu.edu.kz; 6 Department of Emergency Medicine, Semey Medical University, Semey 071407, Kazakhstan; lyudmila.pivina@smu.edu.kz; 7 Department of Scientific Management, National Research Oncology Center, Astana 020000, Kazakhstan; belixina.t@cancercenter.kz; 8 Department of Infectious Diseases, Dermatovenerology, and Immunology, Semey Medical University, Semey 071407, Kazakhstan; saule.maukayeva@smu.edu.kz; 9 Department of Internal Medicine and Rheumatology, Semey Medical University, Semey 071407, Kazakhstan; maiya.goremykina@smu.edu.kz

**Keywords:** antimicrobial resistance, antibiotic consumption, social determinants of health, Kazakhstan, Kyrgyzstan, Tajikistan, Russian Federation, Central Asia, Eastern Europe

## Abstract

**Background/Objectives**: This study aims to analyze antibiotic consumption trends from 2014 to 2023 in Kazakhstan, Kyrgyzstan, Tajikistan, and Russia; forecast future trends up to 2030; and identify key social and economic factors influencing antibiotic use. **Methods**: Data on antibiotic consumption were obtained from the World Health Organization Regional Office for Europe Antimicrobial Consumption Network. Social and economic indicators were sourced from the World Bank DataBank. Of the 86 factors initially considered, 35 were included in the analyses. The forecast modeling of antibiotic consumption trends until 2030 and linear regression analysis to assess associations between antibiotic consumption and its predictors were conducted via SPSS. **Results**: The lowest antibiotic consumption rates were observed in Kazakhstan and Russia, whereas Kyrgyzstan and Tajikistan presented higher rates. The projected antibiotic consumption rates are expected to decline only in Kazakhstan, with other countries remaining stable. Birth and death rates, as well as under-five mortality rates, were significant determinants of antibiotic consumption in Kazakhstan and Tajikistan. In Russia, per capita GDP was a key determinant, whereas in Kazakhstan, inflation in consumer prices played a significant role. Additionally, cereal production was significantly associated with antibiotic consumption in Kazakhstan. In Kyrgyzstan and Russia, measles immunization rates were important determinants, whereas in Kyrgyzstan, access to clean fuels and technologies for cooking was significantly associated with antibiotic consumption. **Conclusions**: The findings of this study provide valuable insights for strengthening antimicrobial stewardship programs by addressing key social and economic determinants of antibiotic use.

## 1. Introduction

Antimicrobial resistance (AMR) is a rising global health problem that contributes significantly to morbidity and mortality. A recent systematic analysis of the Global Burden of Disease indicated that from 1990 to 2021, there was an 80% increase in AMR-associated mortality among adults 70 years of age and older but a 50% decline in AMR-associated mortality among children younger than 5 years of age [1]. Since population aging is a global trend, the burden of AMR is likely to continue growing, with a 70% increase in AMR-associated deaths by 2050 compared with that in 2022 [1]. The inappropriate use of antibiotics is the primary driver of the growing burden of bacterial AMR [2]. This irrational use is influenced by various factors, including inappropriate prescription practices, self-medication, inadequate healthcare infrastructure, limited diagnostic capabilities, a lack of antimicrobial stewardship (AMS) programs, and weak regulatory frameworks that allow over-the-counter (OTC) sales of antibiotics [3]. In addition, different socioeconomic factors contribute to the inappropriate use of antibiotics, further accelerating the spread of bacterial AMR [4].

The concept of social determinants of health (SDHs) has become widely recognized in research as a framework for understanding how nonmedical factors influence health outcomes. The World Health Organization (WHO) defines SDHs as the conditions in which people are born, grow, work, live, and age, as well as the broader forces shaping daily life conditions [5]. Examples of SDHs include but are not limited to economic factors such as unemployment and job insecurity, education, nutrition and behavioral factors, housing and basic amenities, environmental conditions, social inclusion and protection, and access to affordable, high-quality healthcare services [6]. SDH largely reflects health equity and inequity, which refer to the unjust differences in health status observed within and between countries [7]. These disparities exist in all countries, regardless of income level, but they tend to be disproportionately pronounced in low- and middle-income countries (LMICs) [8].

The dissolution of the Union of Soviet Socialist Republics in 1991 led to the formation of 15 independent states across both the European and Asian continents. The Asian post-Soviet countries include Kazakhstan, Kyrgyzstan, Uzbekistan, Tajikistan, and Turkmenistan, all of which share similar socioeconomic conditions, a comparable healthcare system model, and strong historical, political, and economic ties with the region’s hegemonic power—the Russian Federation (hereafter referred to as Russia) [9]. All countries have taken steps to address the growing problem of AMR by developing and implementing national action plans (NAPs), although the effectiveness of these efforts varies significantly [10]. Additionally, most countries in the region report antibiotic consumption data to the WHO Regional Office for Europe [11] and conduct independent studies to monitor antibiotic use [12,13,14,15]. However, to the best of our knowledge, no study has systematically examined the impact of SDH on antibiotic consumption in this group of countries. Therefore, this study aims to address this gap by analyzing antibiotic consumption trends from 2014 to 2023, forecasting future trends up to 2030, and identifying the key SDH factors influencing antibiotic use. The findings of this study could enable improvements in existing AMS programs and contribute to the development of more effective policies to combat AMR in the region.

## 2. Results

### 2.1. The Observed and Projected Antibiotic Consumption Rates in Kazakhstan, Kyrgyzstan, Tajikistan, and Russia

**Figure 1:**
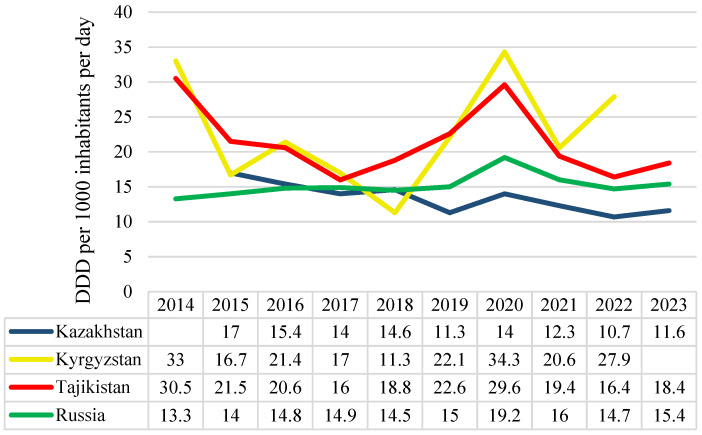
The observed antibiotic consumption rates in Kazakhstan, Kyrgyzstan, Tajikistan, and Russia are expressed in defined daily doses (DDDs) per 1000 inhabitants per day.

During the study period, Kyrgyzstan and Tajikistan had the highest antibiotic consumption rates, whereas Russia and Kazakhstan had the lowest rates. The trend in antibiotic consumption declined in Kazakhstan (average annual percent change (AAPC) = −4.75, 95% confidence interval (CI): −7.23 to −2.21%, *p* = 0.002) and Tajikistan (AAPC = −2.82, 95% CI: −7.96 to 2.61%, *p* = 0.130) but increased in Kyrgyzstan (AAPC = 1.96, 95% CI: −9.06 to 14.31%, *p* = 0.350) and Russia (AAPC = 1.74%, 95% CI: −0.50 to 4.03%, *p* = 0.056). All countries experienced an increase in antibiotic consumption in 2020, which was more pronounced in Kyrgyzstan and Tajikistan. However, by 2021, antibiotic consumption rates had returned to the levels observed in 2019 in all countries studied, except for Kyrgyzstan (Figure 1).

The projections for antibiotic consumption rates until 2030, expressed in defined daily doses per 1000 inhabitants per day (DID), indicate a declining trend only in Kazakhstan. In contrast, the other countries included in this study are expected to maintain stable consumption rates until 2030, at 22.7 DID in Kyrgyzstan, 21.4 DID in Tajikistan, and 15.2 DID in Russia (Table 1).

**Figure 2:**
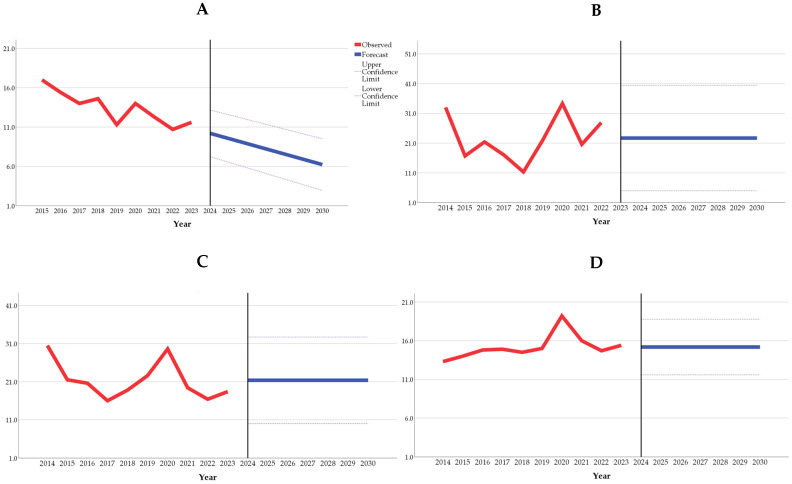
The observed and projected antibiotic consumption rates in Kazakhstan (**A**), Kyrgyzstan (**B**), Tajikistan (**C**), and Russia (**D**), expressed in defined daily doses per 1000 inhabitants per day until 2030.

Figure 2 provides a graphical representation of the observed and projected antibiotic consumption rates (in DID) until 2030, as presented in Table 1. According to the forecast models, antibiotic consumption is expected to decline only in Kazakhstan, whereas in Kyrgyzstan, Tajikistan, and Russia, it is projected to remain stable, with no decreasing trend.

### 2.2. Social Determinants of Health and Antibiotic Consumption in Kazakhstan, Kyrgyzstan, Tajikistan, and Russia

In Kazakhstan, the birth rate, cereal production rate, and under-five mortality rate were positively associated with antibiotic consumption rates, as determined by a linear regression analysis via the enter method. Conversely, GDP per capita, consumer price inflation, and access to clean fuels and technologies for cooking were negatively associated with antibiotic consumption, indicating that an increase in these indicators corresponded to a decline in antibiotic consumption rates. However, performing linear regression with backward elimination resulted in a final model retaining only four significant determinants: birth rate, cereal production, under-five mortality rate (positively associated with antibiotic consumption) and consumer price inflation (negatively associated). The predictive power of both the enter and backward elimination models was strong (R^2^ = 0.985 and 0.949, respectively), although only the backward elimination model achieved statistical significance (Table 2).

In Kyrgyzstan, the rates of measles immunization, access to clean fuels and technologies for cooking, and consumer price inflation were negatively associated with antibiotic consumption rates, as determined by linear regression via the enter method. Conversely, the prevalence of undernourishment, the Gini index, and cereal production were positively associated with antibiotic consumption, indicating that an increase in these indicators corresponded to higher antibiotic consumption rates. The backward elimination model retained only two statistically significant predictors, measles immunization rates and access to clean fuels and technologies for cooking, with the overall model achieving statistical significance (*p* = 0.014) (Table 3).

In Tajikistan, the death rate, incidence of tuberculosis and HIV, livestock production, and proportion of the population aged 0–14 years were positively associated with antibiotic consumption rates, as determined by the enter method. Moreover, GDP per capita was negatively associated with antibiotic consumption. However, the backward elimination method found that the death rate was the only statistically significant predictor with a positive association with antibiotic consumption (Table 4).

In Russia, according to the enter method of linear regression, the measles immunization rates and per capita GDP were negatively associated with antibiotic consumption, whereas unemployment rates, fishery production, and life expectancy at birth were positively associated. However, the backward elimination method retained only measles immunization rates and per capita GDP as significant negative predictors of antibiotic consumption, with an acceptable overall predictive capacity (R^2^ = 0.738, *p* = 0.009) (Table 5).

## 3. Discussion

### 3.1. Antibiotic Consumption Surveillance

The WHO Regional Office for Europe collects and publishes annual reports on antibiotic consumption in non-European Union (EU) countries within the WHO European Region. According to the latest report released in 2024, Turkey had the highest rate of antibiotic consumption among non-EU countries, reaching 42.7 DID in 2023. Serbia and Montenegro reported 31.4 DIDs and 31.0 DIDs, respectively, which are higher than the rates reported in the countries analyzed in this study. Other non-EU countries with higher antibiotic consumption rates than those in this study include Bosnia and Herzegovina (24.7 DID), Georgia (23.7 DID), and Belarus (19.8 DID), as of 2023 [16]. Conversely, certain non-EU countries reported lower antibiotic consumption, including Switzerland (11.0 DID) and Ukraine (10.1 DID), in 2023 [16]. A geographical trend emerges among non-EU countries, with higher antibiotic consumption observed in southern countries such as Turkey, Serbia, Montenegro, Bosnia and Herzegovina, and Georgia. A similar pattern is evident in the two study countries with the highest antibiotic consumption, Tajikistan and Kyrgyzstan, which are geographically located further south than the other study countries are. These nations also have lower per capita GDP levels and are classified as lower-middle-income countries according to the World Bank (WB).

A comparable pattern is observed among EU-member states, where southern European countries tend to have higher antibiotic consumption rates. According to the European Centre for Disease Prevention and Control, in 2023, the highest antibiotic consumption rates in the EU were reported in Greece (28.5 DID), Romania (27.4 DID), and Bulgaria (26.3 DID) [17]. These countries also have lower per capita GDP levels, aligning with the WB classification [18]. In contrast, the EU countries with the lowest antibiotic consumption rates include the Netherlands (9.6 DID), Austria (11.3 DID), Estonia (12.7 DID), and Finland (12.9 DID) [17]. The higher antibiotic consumption rates in certain countries have historically been associated with challenges such as self-medication and OTC access without prescriptions, which are often linked to the weaker enforcement of regulations and public health policies [19].

All countries in this study have established antimicrobial consumption (AMC) surveillance systems with up-to-date NAPs, where antibiotic consumption surveillance is a strategic objective [20]. However, the sources of data on antibiotic consumption vary across countries. Kyrgyzstan tracks antibiotic consumption via import and sales records, Tajikistan relies on import and certification records, Russia uses only sales records, and the data source for Kazakhstan remains unclear [21]. According to the Global Database for Tracking AMR Country Self-Assessment Survey (TrACSS), all countries in this study utilize antibiotic consumption data to inform decision-making and modify policies as needed [22]. Furthermore, the TrACSS reports that all countries have implemented laws and regulations governing the prescription and sale of antimicrobials, which legally prohibit OTC sales [22]. However, recent real-world studies indicate that OTC antibiotic sales persist despite these regulations. For example, in Tajikistan, 21% of respondents reported acquiring antibiotics without a prescription from a pharmacy, whereas in Kyrgyzstan and Kazakhstan, these proportions were higher at 37% and 28%, respectively [23]. In Russia, certain professional groups with access to antibiotics, such as pharmacists, tend to self-medicate [24].

An increase in antibiotic consumption during the COVID-19 pandemic, particularly in 2020, was observed in all countries under study. This surge may be attributed to the initial search for effective treatments against COVID-19, during which certain antibiotics—such as azithromycin—were believed to possess antiviral properties. As a result, these antibiotics are sometimes used inappropriately for the management of COVID-19 and its potential secondary bacterial infections [25].

### 3.2. Social Determinants of Health and Antibiotic Consumption

Population demographics can influence antibiotic consumption in a variety of ways. A high birth rate increases the size of the pediatric population, leading to a higher prevalence of childhood infections that often require antibiotic treatment [26]. Moreover, in LMICs, under-five children are frequently overmedicated with antibiotics [27], and these antibiotics are sometimes obtained from unregulated or unqualified sources [28]. In this study, the birth rate was positively associated with antibiotic consumption in Kazakhstan (standardized beta = 0.540, *p* = 0.029). This finding may be partially explained by the country’s high vaccine hesitancy rates [29], which could contribute to an increased burden of vaccine-preventable infections and, consequently, greater antibiotic use. In addition, in Kazakhstan, antibiotic consumption was significantly associated with under-five mortality (standardized beta = 0.685, *p* = 0.005). This association may suggest that critically ill pediatric patients receive extensive antibiotic treatment [30], but further studies are needed to confirm this hypothesis. Similarly, in Tajikistan, the death rate was significantly associated with antibiotic consumption (standardized beta = 0.703, *p* = 0.023). Higher mortality rates are often linked to an increased burden of infectious diseases, potentially driving increased antibiotic use [31]. Another plausible explanation is that critically ill patients in Tajikistan may receive excessive antibiotic treatment [32], possibly as a last-resort measure in severe cases. While the findings of this study highlight potential demographic drivers of antibiotic consumption in Central Asia, further research is needed to establish causality and explore the underlying mechanisms.

Economic factors were another key determinant of antibiotic consumption in the countries under study. In particular, per capita GDP exhibited a significant negative association with antibiotic use in Russia (Standardized Beta = −0.682, *p* = 0.018). Globally, antibiotic consumption in LMICs has been shown to increase with GDP growth, sometimes even surpassing consumption levels in high-income countries [33]. However, Russia is classified as a high-income country by the WB, and the observed decline in antibiotic consumption with GDP growth could be attributed to several factors: improved access to healthcare services, a greater availability of trained medical professionals, the stricter enforcement of prescription regulations, enhanced AMR surveillance, and a reduced burden of infectious diseases [34]. Compared with other countries in this study, Russia has higher per capita rates of physicians and hospital beds [9] and has also established a standardized national AMR surveillance system [10], which may explain the negative association between GDP and antibiotic consumption. Another economic factor, inflation in consumer prices, was significantly negatively associated with antibiotic consumption in Kazakhstan (Standardized Beta = −0.435, *p* = 0.031). Kazakhstan’s healthcare system requires substantial out-of-pocket expenditures from patients [35]. As inflation increases the cost of medications, patients may adopt more selective purchasing behaviors, leading to a decline in antibiotic consumption [36].

Among all the agriculture-related determinants tested (cereal, livestock, and fisheries production), cereal production was significantly associated with antibiotic consumption in only Kazakhstan, and this association was positive. Cereal production can contribute to an increase in antibiotic consumption through several mechanisms. Large-scale cereal production is often linked to livestock farming, as cereals are primary components of animal feed; greater livestock production, in turn, can lead to increased antibiotic use in food-producing animals [37]. In addition, crop production is commonly associated with the use of manure as fertilizer, and antibiotic-containing manure can lead to the accumulation of antibiotics in cereals [38]. Moreover, intensive cereal farming may result in increased exposure to pesticides, fertilizers, and other agrochemicals, which could indirectly impact human health and drive increased antibiotic use to manage associated health conditions [39]. Kazakhstan is the leading cereal producer in Central Asia, with its cereal exported to neighboring countries [40]. Given these agricultural developments, adherence to the One Health approach is essential to ensure proper antibiotic regulation across the human, animal, and environmental sectors [41]. However, there are shortcomings in the effective integration of the One Health approach, particularly in regulatory oversight, surveillance, and multisector coordination [10].

Air pollution has many adverse effects on human health, with the respiratory tract being the primary site of impact. Even short-term exposure to air pollution worsens respiratory symptoms, often leading to increased antibiotic prescriptions [42]. The harmful effects of air pollution are commonly attributed to pollutants such as particulate matter ≤ 10 μm in diameter (PM10), PM2.5, nitrogen dioxide, and ozone [43]. Although the WB DataBank provides data on these pollutants for all countries under study [18], they were excluded from the present analysis because of incomplete data availability, with 2020 being the most recent year reported. Access to clean fuels and technologies for cooking was the only up-to-date air quality variable included in the analysis, showing a significant negative association with antibiotic consumption in Kyrgyzstan (Standardized Beta = −0.788, *p* = 0.012). Since air pollution is a major public health concern in the region [44,45,46], future studies should explore its role in antibiotic consumption, particularly its impact on respiratory and other pollution-related health conditions.

Immunization plays a critical role in reducing antibiotic consumption by preventing infectious diseases that would otherwise require antibiotic treatment. The beneficial effects were demonstrated not only for bacterial infections but also for viral infections, for which antibiotics are often misused [47]. The present study revealed a significant negative association between antibiotic consumption and measles immunization in Kyrgyzstan (standardized beta = −0.815, *p* = 0.010) and Russia (standardized beta = −0.953, *p* = 0.004). However, vaccine hesitancy remains a major public health challenge in the countries under study and has contributed to periodic disease outbreaks [48]. Addressing vaccine hesitancy, particularly for pediatric infections, is essential not only to reduce the incidence of vaccine-preventable diseases but also to mitigate antibiotic misuse and combat AMR [49].

### 3.3. Implications for Public Health Policy

The findings of this study offer important insights for shaping public health policy across the countries under study. Given the heterogeneous patterns of antibiotic consumption observed across countries—and their associations with distinct SDHs—a one-size-fits-all policy approach is unlikely to be effective. Instead, national and regional policies should be tailored to the specific SDH profiles and health system contexts of each country. First, the persistently high antibiotic consumption rates in Kyrgyzstan and Tajikistan call for urgent policy interventions to strengthen the enforcement of existing antimicrobial regulations. Although all the study countries formally prohibit OTC antibiotic sales, real-world data highlight widespread noncompliance [11]. Policymakers should invest in improving regulatory oversight, scaling up pharmacy audits, increasing penalties for violations, and strengthening community-based awareness campaigns about the risks of self-medication [50]. Second, the study revealed that demographic factors such as high birth rates and under-five mortality significantly influence antibiotic use, particularly in Kazakhstan and Tajikistan. These findings highlight the need for pediatric-specific interventions, including the promotion of rational antibiotic prescribing practices in maternal and child health services and the integration of AMR content into pediatric care guidelines and training programs [51].

Additionally, immunization coverage has emerged as a strong negative predictor of antibiotic consumption, particularly in Kyrgyzstan and Russia. Policymakers should prioritize vaccination campaigns, especially in communities with high vaccine hesitancy. Targeted communication and outreach efforts, supported by local health professionals, can enhance public trust in vaccines and help counteract misinformation [52]. Finally, these findings emphasize the necessity of cross-sectoral collaboration and multilevel governance. The Ministries of Health (MoH), Agriculture, Environment, and Education must work together to design coherent AMR action plans that are grounded in data, the context, and are regularly updated [41].

### 3.4. Study Limitations

This study has several limitations that must be acknowledged. The primary limitation is its ecological design, which prevents the establishment of causal relationships. However, analyzing a large dataset with diverse variables provided valuable insights into the potential contributions of factors outside the healthcare sector to antibiotic consumption. Another limitation is the limited availability of many variables that could serve as potential determinants of antibiotic consumption, particularly those related to education and social protection. Additionally, data on antibiotic consumption were available only for a ten-year period for Russia and Tajikistan (and for Kazakhstan and Kyrgyzstan, only for nine years), which may not be sufficient to capture long-term trends or fully assess the impact of the investigated SDH on antibiotic use. In addition, data on antibiotic consumption were entirely unavailable for Turkmenistan and were only available for four years in Uzbekistan, preventing the inclusion of these countries in the analysis.

Additionally, the European Office of the WHO collects antibiotic consumption data from different types of sources across countries in the region. As a result, the observed trends in antibiotic consumption may be influenced by inconsistencies in data collection methods. The short time frame also restricts our ability to evaluate lagged effects, which are likely to exist between antibiotic consumption and certain predictors, such as immunization coverage, agricultural production, and air pollution. Future studies should aim to incorporate a longer time series and a broader range of variables to increase the robustness of the findings and better understand the complex drivers of antibiotic consumption in Central Asia and Russia.

## 4. Materials and Methods

### 4.1. Countries Under Study

**Figure 3:**
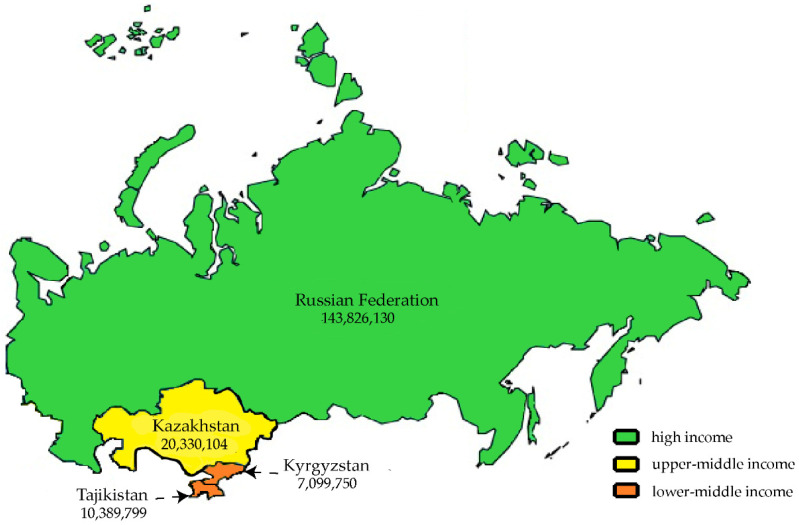
Map of countries included in the study, indicating the income level according to the World Bank and the population size as of 2023.

Central Asia comprises a group of five countries: Kazakhstan, Kyrgyzstan, Uzbekistan, Tajikistan, and Turkmenistan. The WB classifies two of these countries—Kazakhstan and Turkmenistan—as upper-middle-income countries and three other countries—Kyrgyzstan, Uzbekistan, and Tajikistan—as lower-middle-income countries [18]. All countries have systems in place to monitor antibiotic consumption [22]. However, only Kazakhstan, Kyrgyzstan, and Tajikistan reported antibiotic consumption data to the WHO Regional Office for Europe on a regular basis, while Uzbekistan only reported data for the period of 2016–2019, and Turkmenistan did not report data at all [16]. Therefore, for the purpose of this study, data on antibiotic consumption from Kazakhstan, Kyrgyzstan, and Tajikistan were included in the analysis. All comparisons were made with Russia, for which antibiotic consumption data are available for the entire period (2014–2023). Figure 3 presents a map of the countries under study, showing their income levels on the basis of the WB classification and their population sizes as of 2023 [18].

### 4.2. Data Sources

The WB DataBank served as the primary data source for this study. This database provides a comprehensive collection of accurate time series data on various topics, including economic indicators, demographic trends, health statistics, and environmental factors, compiled from officially recognized international sources [18]. Additionally, data on antibiotic consumption from the WHO Regional Office for Europe AMC Network were utilized. The WHO Regional Office for Europe publishes annual reports on antibiotic consumption in non-European Union countries within the WHO European Region [16]. In addition, official statistical compilations on health indicators issued by the MoH of the countries under study were also utilized as supplementary data sources [53,54].

### 4.3. Study Variables

The annual rates of antibiotic consumption, expressed in DID, served as the primary study variable and were used as the outcome variable. These data were extracted from the WHO Regional Office for Europe annual reports on AMC [16]. The predictor variables were selected on the basis of previous studies [55,56,57] and encompassed various domains, including health, healthcare services and expenditure, sanitation and hygiene, education, air pollution, agriculture, economic development and equity, employment and social protection, population dynamics, nutrition, and behavior. The WB DataBank [18] served as the primary source for these variables, which were systematically reviewed for suitability in this study. The main exclusion criterion was limited data availability, specifically cases where data were only partially available for the study period (2014–2023). In addition, data on healthcare services—particularly indicators related to the availability of physicians, nurses, and other healthcare professionals, as well as hospital bed capacity—were obtained from statistical reports issued by the MoH [53,54]. Of the 86 variables initially considered, 35 met the inclusion criteria and were incorporated into the analysis. A comprehensive list of included and excluded variables, along with the reasons for non-inclusion, is provided in the Supplementary Materials (Supplementary Tables S1 and S2).

### 4.4. Data Analysis

All extracted variables were organized into Excel spreadsheets, which are provided in the Supplementary Materials. Separate spreadsheets were created for the DID in each country under analysis to assess time trends, as well as for both predictor and outcome variables to analyze associations. All the statistical analyses were conducted via the Statistical Package for the Social Sciences (SPSS, version 24.0; Armonk, NY, USA). A 5% Type I error level was used to determine the statistical significance (*p* < 0.05).

Time series analyses were performed to examine historical trends over the study period and to generate forecasts until 2030. The AAPC was calculated along with its 95% CI to evaluate past trends. The forecasting of future trends was performed via the Expert Modeler function in SPSS, which automatically identified the best-fitting epidemiological model. Projected estimates, along with corresponding plots, were generated and included in the report. The parameters of the selected model, along with its associated *p* value, are also reported.

The analysis of the associations between predictor variables and antibiotic consumption rates was conducted in several steps. First, only predictor variables with no more than 10% missing values were included in the analysis (i.e., at most one missing value per variable). Any missing values were imputed by calculating the mean of the remaining time points. To address multicollinearity, Pearson’s correlation analysis was conducted for all the study variables. Predictor variables with high correlation coefficients (r ≥ 0.8) were considered prone to multicollinearity, and the decision on which variable to retain for further analysis was based on its correlation strength with the outcome variable (DID), with the strongest predictor being retained. Linear regression analysis was then performed via both the enter method and backward elimination method. The backward elimination method removes variables iteratively on the basis of a predefined significance threshold (*p* < 0.05), ensuring that only significant predictors are retained in the final model. Only standardized coefficients (Beta) along with the lower and upper bounds of the 95% CI are reported. The predictive capacity of the regression model was assessed via the coefficient of determination (R^2^) and reported along with the model’s p value. Statistical significance was interpreted as follows: *p* < 0.05 was considered statistically significant, *p* < 0.01 was considered highly significant, and *p* < 0.001 was considered highly significant.

## 5. Conclusions

There was a disparity in antibiotic consumption among the countries included in this study, with larger and wealthier countries (Kazakhstan and Russia) exhibiting lower consumption rates. Except for Kazakhstan, none of the countries demonstrated a potential decline in antibiotic consumption by 2030, highlighting the need for the broader implementation of AMS programs. Several SDHs were found to be significantly associated with antibiotic consumption across the region, including population demographics (birth and death rates, underfive mortality), economic factors (per capita GDP and inflation of consumer prices), agriculture (cereal production), air pollution (access to clean fuels and technologies for cooking), and measles immunization coverage. Prospective studies are needed to better understand both the healthcare- and non-healthcare-related factors influencing antibiotic consumption in each country, enabling the development of more tailored and effective AMS strategies.

## Figures and Tables

**Table 1:** Projected antibiotic consumption rates in Kazakhstan, Kyrgyzstan, Tajikistan, and Russia, expressed in defined daily doses per 1000 inhabitants per day until 2030.

Year	Country			
	Kazakhstan	Kyrgyzstan	Tajikistan	Russia
2024	10.2	22.7	21.4	15.2
2025	9.5	22.7	21.4	15.2
2026	8.9	22.7	21.4	15.2
2027	8.2	22.7	21.4	15.2
2028	7.6	22.7	21.4	15.2
2029	6.9	22.7	21.4	15.2
2030	6.2	22.7	21.4	15.2
Model parameters	Holt, *p* = 0.509	ARIMA (0.0.0) *p* < 0.001 ^+^	ARIMA (0.0.0) *p* < 0.001 ^+^	ARIMA (0.0.0) *p* < 0.001 ^+^

^+^ The association was statistically highly significant.

**Table 2:** Multivariable associations between antibiotic consumption rates and predictors in Kazakhstan, 2015–2023.

Predictors	Model 1 (Enter Method)		Model 2 (Backward Method)	
	Standardized Coefficients (95% Confidence Interval)	*p* Value	Standardized Coefficients (95% Confidence Interval)	*p* Value
Birth rate, crude	0.509 (−0.888; 3.417)	0.127	0.540 (0.221; 2.459)	0.029 *
GDP per capita	−0.192 (−0.001; 0.001)	0.351	-	-
Inflation, consumer prices	−0.384 (−0.581; 0.184)	0.155	−0.435 (−0.416; −0.034)	0.031 *
Access to clean fuels and technologies for cooking	−0.106 (−13.931; 10.265)	0.581	-	-
Cereal production	0.636 (0.000; 0.000)	0.095	0.705 (0.000; 0.000)	0.015 *
Mortality rate, under 5	0.650 (0.188; 4.004)	0.042 *	0.685 (1.119; 3.294)	0.005 °
Model parameters	R^2^ = 0.971	0.085	R^2^ = 0.949	0.008 °

* The association was statistically significant. ° The association was statistically very significant.

**Table 3:** Multivariable associations between antibiotic consumption rates and predictors in Kyrgyzstan, 2014–2022.

Predictors	Model 1 (Enter Method)		Model 2 (Backward Method)	
	Standardized Coefficients (95% Confidence Interval)	*p* Value	Standardized Coefficients (95% Confidence Interval)	*p* Value
Immunization, measles	−1.055 (−9.595; 1.948)	0.104	−0.815 (−4.909; −1.000)	0.010 *
Prevalence of undernourishment	0.210 (−30.192; 36.313)	0.730	-	-
Access to clean fuels and technologies for cooking	−1.047 (−45.708; 8.281)	0.096	−0.788 (−23.722; −4.443)	0.012 *
Inflation, consumer prices	−0.244 (−3.709; 2.923)	0.661	-	-
Gini index	0.260 (−6.683; 10.009)	0.482	-	-
Cereal production	0.344 (0.000; 0.000)	0.327	-	-
Model parameters	R^2^ = 0.897	0.279	R^2^ = 0.758	0.014 *

* The association was statistically significant.

**Table 4:** Multivariable associations between antibiotic consumption rates and predictors in Tajikistan, 2014–2023.

Predictors	Model 1 (Enter Method)		Model 2 (Backward Method)	
	Standardized Coefficients (95% Confidence Interval)	*p* Value	Standardized Coefficients (95% Confidence Interval)	*p* Value
Death rate, crude	0.833 (0.394; 18.004)	0.045 *	0.703 (1.358; 14.162)	0.023 *
Incidence of HIV	0.863 (−558.161; 1617.036)	0.219	-	-
Incidence of tuberculosis	1.556 (−0.500; 4.799)	0.082	-	-
Livestock production	1.109 (−0.129; 0.406)	0.198	-	-
GDP per capita	−0.121 (−0.044; 0.042)	0.954	-	-
Population ages 0–14	1.577 (−17.142; 137.327)	0.090	-	-
Model parameters	R^2^ = 0.871	0.172	R^2^ = 0.494	0.023 *

* The association was statistically significant.

**Table 5:** Multivariable associations between antibiotic consumption rates and predictors in Russia, 2014–2023.

Predictors	Model 1 (Enter Method)		Model 2 (Backward Method)	
	Standardized Coefficients (95% Confidence Interval)	*p* Value	Standardized Coefficients (95% Confidence Interval)	*p* Value
Immunization, measles	−1.043 (−6.745; 0.322)	0.065	−0.953 (−4.551; −1.319)	0.004 °
GDP per capita	−0.409 (−0.001; 0.000)	0.302	−0.682 (−0.001; 0.000)	0.018 *
Unemployment, total	0.429 (−1.327; 3.199)	0.315	-	-
Total fisheries production	0.071 (0.000; 0.000)	0.844	-	-
Life expectancy at birth	0.283 (−0.991; 1.910)	0.429	-	-
Model parameters	R^2^ = 0.827	0.109	R^2^ = 0.738	0.009 °

* The association was statistically significant. ° The association was statistically very significant.

## Data Availability

The data presented in this study are provided in the Supplementary Materials.

## Supplementary Materials

The following supporting information can be downloaded at https://www.mdpi.com/article/10.3390/antibiotics14050513/s1, Table S1: Data sources for indicators included in the analysis; Table S2: Data sources for indicators not included in the analysis and the reasons for noninclusion.

## Abbreviations

The following was used in this manuscript:

AAPS	Average Annual Percent Change
AMR	Antimicrobial Resistance
AMS	Antimicrobial Stewardship
DID	Defined Daily Doses per 1000 Inhabitants per Day
EU	European Union
GDP	Gross Domestic Product
HIV	Human Immunodeficiency Virus
LMICs	Low- and Middle-Income Countries
MoH	Ministry of Health
NAP	National Action Plan
OTC	Over-the-Counter
PM	Particulate Matter
SDH	Social Determinants of Health
SPSS	Statistical Package for the Social Sciences
TrACSS	Tracking AMR Country Self-Assessment Survey
WB	World Bank
WHO	World Health Organization
USA	United States of America
95% CI	95% Confidence Interval
