# Supplementary material for: Social Determinants of Health and Antibiotic Consumption
Source: Antibiotics (Basel). 2025 May 15;14(5):513. doi: 10.3390/antibiotics14050513 (PMC12108191; doi:10.3390/antibiotics14050513)
Supplement: Supplementary file 1 [file antibiotics-14-00513-s001.zip › antibiotics-3568388-supplementary.pdf]

Table S1. Data sources for indicators included in the analysis.

	Indicators	Data source	Years of data availability
Health and healthcare services	Defined Daily Doses (per 1,000 population per day)	World Health Organization	2014-2023
	Tuberculosis incidence rate (per 100,000 people)	World Bank DataBank	
	Mortality rate, neonatal (per 1,000 live births)		2014-2022
	Mortality rate, under-5 (per 1,000 live births)		
	Immunization, measles (% of children ages 12-23 months)		2014-2023
	Immunization, DPT* (% of children ages 12-23 months)		
	Vitamin A supplementation coverage rate (% of children ages 6-59 months)		2014-2022 (Tajikistan only)
	Hospital beds (per 1,000 people)		2014-2023 (for Kazakhstan Kyrgyzstan only)
	Prevalence of undernourishment (% of population)		2014-2022
	Physicians (per 1,000 people)	National Statistical Compilations	2015-2023 (for Kazakhstan only)
	Nurses and dentists		
Sanitation and hygiene	People using at least basic sanitation services (% of population)	World Bank DataBank	2014-2022
	People using safely managed sanitation services (% of population)		
	People practicing open defecation (% of population)		
	People using at least basic drinking water services (% of population)		
Air pollution	Access to clean fuels and technologies for cooking (% of population)		2014-2022
Agriculture	Cereal production (metric tons)		
	Total fisheries production (metric tons)		
	Livestock production index		
Economic development and equity	GDP© per capita (current US$∞)	World Bank DataBank	2014-2023
	Inflation, consumer prices (annual %)		
	Gini index		2014-2022 (except for Kazakhstan)
Employment	Unemployment, total (% of total labor force)	International Labor Organization	
Health expenditure	Current health expenditure per capita (current US$)	World Bank DataBank	2014-2021 (Kyrgyzstan only)
Population	Population ages 65 and above (% of total population)	World Bank DataBank	2014-2023
	Population ages 0-14 (% of total population)		
	Rural population (% of total population)		
	Population growth (annual %)		
	Birth rate, crude (per 1,000 people)		2014-2022
	Death rate, crude (per 1,000 people)		
	Life expectancy at birth, total (years)		
	Refugee population by country or territory of asylum		2014-2023
Behaviour	Incidence of HIV, all (per 1,000 uninfected population)		2014-2022 (for Tajikistan and Kyrgyzstan only)

*Diphtheria, pertussis, tetanus; ©GDP, gross domestic product; ∞United States Dollars.

Table S2. Data sources for indicators not included in the analysis and the reasons for non-inclusion.

	Indicators	Data source	Years of data availability	Reason for non-inclusion
Health and healthcare services	Pregnant women receiving prenatal care (%)	World Bank DataBank	2015, 2018	Limited data availability
	Number of surgical procedures (per 100,000 population)		2016, 2020	
	Incidence of malaria (per 1,000 population at risk)		-	Non-occurrence
	Newborns protected against tetanus (%)			Non-existence of data
	Community health workers (per 1,000 people)			
	Specialist surgical workforce (per 100,000 population)			
	Cause of death, by injury (% of total)		2015, 2019	Limited data availability
	Cause of death, by non-communicable diseases (% of total)			
	Cause of death, by communicable diseases and maternal, prenatal and nutrition conditions (% of total)			
	Diabetes prevalence (% of population ages 20 to 79)		2021	
	UHC* service coverage index		2015, 2017, 2019, 2021	
	Births attended by skilled health staff (% of total)		Separate years only, depending on the country	
	Prevalence of severe wasting, weight for height (% of children under 5)			
	Prevalence of stunting, height for age (% of children under 5)			
	Diarrhea treatment (% of children under 5 receiving oral rehydration and continued feeding)			
	ARI** treatment (% of children under 5 taken to a health provider)			
	Exclusive breastfeeding (% of children under 6 months)			
	Vitamin A supplementation coverage rate (% of children ages 6-59 months)		Unavailability of data, except for Tajikistan	
	Antiretroviral therapy coverage (% of people living with HIV)		Unavailability of data, except for Tajikistan and Kyrgyzstan	
	Prevalence of anemia among pregnant women (%)		2014-2019	Limited data availability
	Prevalence of anemia among non-pregnant women (% of women ages 15-49)			
	Prevalence of anemia among children (% of children ages 6-59 months)			
	Nurses and beds		2014-2020	
	Maternal mortality ratio (modeled estimate, per 100,000 live births)			
	Low-birthweight babies (% of births)		2014-2020	
Education	Educational attainment, at least completed primary, population 25+ years, total (%) (cumulative)	World Bank DataBank	Separate years only, depending on the country	Limited data availability
	Educational attainment, at least completed lower secondary, population 25+, total (%) (cumulative)			
	Educational attainment, at least completed upper secondary, population 25+, total (%) (cumulative)			
	Educational attainment, at least Bachelor's or equivalent, population 25+, total (%) (cumulative)			
	Educational attainment, at least Master's or equivalent, population 25+, total (%) (cumulative)			
	School enrollment, secondary (% net)			
	Compulsory education, duration (years)		2014-2023	Constant time series
Nutrition	Prevalence of severe food insecurity in the population (%)		Separate years only, depending on the country	Limited data availability

Social protection	Coverage of social insurance programs in 3rd quintile (% of population)	2015, 2017, 2019, 2020	
	Benefit incidence of social insurance programs to poorest quintile (% of total social insurance benefits)	2015, 2017, 2018, 2020	
	Coverage of unemployment benefits and ALMP*** in 3rd quintile (% of population)		
	Adequacy of social protection and labor programs (% of total welfare of beneficiary households)		
	Coverage of social insurance programs (% of population)	2014, 2015	
	Adequacy of social protection and labor programs (% of total welfare of beneficiary households)	Separate years only, depending on the country	
	Proportion of population pushed below the $3.65 ($ 2017 PPP[©]) poverty line by out-of-pocket health care expenditure (%)		
	Proportion of population pushed further below the $2.15 ($ 2017 PPP) poverty line by out-of-pocket health care expenditure (%)		
Air pollution	PM2.5[∞] air pollution, population exposed to levels exceeding WHO guideline value (% of total)	2014-2017	
	PM2.5 air pollution, mean annual exposure (micrograms per cubic meter)	2014-2020	
	Total greenhouse gas emissions including LULUCF[Ω] (Mt CO2e)		
Sanitation and hygiene	People with basic handwashing facilities including soap and water (% of population)	Separate years only, depending on the country	
Composite	Multidimensional poverty headcount ratio (World Bank) (% of population)		
	Human capital index (scale 0-1)	2017,2018,2020	
Population	International migrant stock (% of population)	2015	
	Population living in slums (% of urban population)	2016,2018,2020	
Economic development	Poverty headcount ratio at national poverty lines (% of population)	Separate years only, depending on the country	
Behaviour	Prevalence of current tobacco use (% of adults)		
	Incidence of HIV[0], all (per 1,000 uninfected population)	Unavailability of data, except for Tajikistan and Kyrgyzstan	
	Total alcohol consumption per capita (liters of pure alcohol, projected estimates, 15+ years of age)	2014-2020	Limited data availability
Health expenditure	Proportion of population spending more than 10% of household consumption or income on out-of-pocket health care expenditure (%)	Separate years only, depending on the country	Limited data availability

*Universal Health Coverage, **Acute Respiratory Infection, *** Active Labor Market Policies, [©]Purchasing Power Parity, [0]Human Immunodeficiency Virus, [∞] Particulate matter with a diameter of 2.5 micrometres; [Ω] Land Use, Land-Use Change, and Forestry,.
